# The Kidney in Heart Failure: The Role of Venous Congestion

**DOI:** 10.14797/mdcvj.1121

**Published:** 2022-09-06

**Authors:** Alvaro Tamayo-Gutierrez, Hassan N. Ibrahim

**Affiliations:** 1 Division of Renal Diseases and Hypertension, Houston Methodist Hospital, Houston, Texas, US; 2 Division of Kidney Diseases, Hypertension & Transplantation, McGovern Medical School, University of Texas Health Science Center at Houston, Houston, Texas, US

**Keywords:** heart failure, cardiorenal syndrome, venous congestion, glomerular filtration rate

## Abstract

Heart failure can lead to renal impairment, an interaction now termed “cardiorenal syndrome.” The prevalent physiological explanation for the renal impairment that accompanies heart failure centers around the forward failure hypothesis, which emphasizes the role of left ventricular dysfunction in causing edema, and the backward failure hypothesis, which singles out venous congestion as the dominant mechanism of edema and reduced glomerular filtration rate. In this review, we provide an appraisal on venous congestion, an extremely important contributor that has received little attention. We also summarize the pharmacology of loop diuretics, explain current understanding of diuretic resistance, and address controversies regarding decongestive treatments.

## Introduction

Acute worsening of renal function accompanies acute decompensated heart failure in nearly one-third of cases and not only leads to prolonged hospitalization but also is an independent risk factor of mortality.^1,2^ The concept of cardiorenal syndrome has gained popularity in the literature in recent years and is now used to describe five different sequential bidirectional causal relationships between these two organs (Table 1).^3^ It is important to remember, however, that these two organs commonly share risk factors, such as hypertension and diabetes, that can independently cause organ dysfunction.

**Table 1:** Types of cardiorenal syndromes. Adapted from Acute Dialysis Quality Initiative consensus group, 2010.


SYNDROME	TYPE	DEFINITION

Acute cardiorenal	1	Acute worsening of heart function leading to acute kidney injury

Chronic cardiorenal	2	Chronic heart failure leading to kidney injury or dysfunction

Acute renocardiac	3	Acute kidney injury leading to heart failure

Chronic renocardiac	4	Chronic kidney disease leading to heart failure

Secondary cardiorenal	5	Systemic conditions leading to simultaneous injury


Most agree that acute decompensated left-sided heart failure with left ventricular (LV) failure can lead to a decrease in glomerular filtration rate (GFR), particularly when mean arterial pressure falls below the threshold of renal autoregulation. Yet recent observations suggest that elevated central venous pressure is a major predictor of worse renal function independent of LV function. For over a century, physicians and physiologists have used a variety of animal models and human studies to attempt to answer the question of whether venous congestion can lead to a decline in GFR. This is a highly relevant clinical question because the nephrologist is commonly consulted to provide “decongestion” in patients with high right-sided venous pressure in the setting of venous congestion and preserved LV function. Herein, we provide a detailed appraisal of the current physiological understanding of the impact of elevated central venous pressure (CVP) and consequently elevated renal venous pressure (RVP) on renal hemodynamics, address possible mechanisms by which venous congestion can lead to a decline in GFR, and review current decongestive strategies.

## Forward Versus Backward Failure Hypothesis

The kidneys receive about 20% of the cardiac output. This is a massive allocation relative to total renal mass and metabolic needs. Notably, only about 20% of plasma flow to the kidney is actually filtered into Bowman’s space; therefore, the kidney can significantly alter renal blood flow (RBF) without compromising metabolic needs. However, RBF is only one of many factors that can alter GFR, as the latter is the product of net filtration pressure and the filtration coefficient.

Elevated CVP, regardless of the cause but in the presence of volume overload, is a robust hemodynamic predictor of acute worsening in renal function.^4^ The decline in renal function in the setting of venous congestion and right heart failure with normal LV function has been referred to as “backward failure.” There has been much debate over the contribution of “forward failure” versus “backward failure” to the worsening in renal function seen in heart failure at both the clinical and experimental level. The mechanism by which systolic dysfunction can lead to a decrease in GFR is rather intuitive. Once RBF falls below the threshold for renal autoregulation, GFR declines. This “forward failure” theory has been the prevailing theory for renal dysfunction in the setting of heart failure for over a century. Merrill and colleagues demonstrated that a low cardiac index is associated with a decline in renal plasma flow (RPF) and GFR, but recent human studies indicate that LV dysfunction does not appear to be a predictor of worsening renal function until cardiac output becomes severely depressed.^4,5,6^ Moreover, about half of patients with decompensated heart failure have preserved LV function.^7^

### Contribution of Venous Congestion to GFR Reduction

Several lines of evidence support that elevated CVP is transmitted to the renal vein, resulting in elevation of RVP that can lead to a reduction in RBF. Winton et al., utilizing a dog isolated perfused heart-lung-kidney model, found a significant decline in RBF when RVP was increased to 24 mm Hg (while mean arterial pressure was held constant).^8^ In an attempt to address how this occurs, Corradi et al. found that elevated RVP in a rat led to a decrease in RBF and a rise in renal venous resistance in innervated kidneys.^9^ In denervated kidneys, however, the decrease in renal venous resistance was less pronounced, suggesting that active participation of the sympathetic nervous system appears to be a prerequisite for this to occur.

Maxwell et al. measured renal hemodynamics, including RVP, in humans with and without heart failure.^10^ In normal subjects, RVP ranged from 10 mm Hg to 15 mm Hg with an average of 11 mm Hg. In those with heart failure, RVP ranged from 13 mm Hg to 30 mm Hg with an average of 13.5 mm Hg. In heart failure patients, renal resistance was elevated to 172% that of controls. The same investigators substituted an elevated RVP of 22 mm Hg seen in heart failure for a control value of 10 mm Hg to calculate RBF in normal subjects and found that RBF went from 1200 mL/min to 1032 mL/min, a decrease of only 14%.

Whether elevated RVP causes a rise in renal interstitial pressure (RIP) and/or renal intratubular pressure is a critical question, as elevation in the RIP could be transmitted back up to the tubular system and, if the intratubular pressure rises sufficiently to oppose the intraglomerular hydrostatic pressure GFR, may not only decline but cease altogether. Ludwig et al. suggested in the 1860sI that a rise in RVP may indeed lead to a rise in RIP and a decline in GFR.^11^ The significance of elevated RIP in response to elevated RVP was discussed further by Winton et al.^12,13^ RIP was assessed through direct measurement by introducing a needle into the renal parenchyma after a hypovolemic state was induced by venesection, which resulted in a fall in renal interstitial pressure. Giving noradrenaline and adrenaline led to an increase in mean arterial pressure but resulted in a decrease in RIP. They proposed that RIP changes were related to alterations in intrarenal vascular tone as well as the pressure difference across the kidney, ie, the arteriovenous (AV) gradient. Overall, the consensus is that elevated RVP does lead to a rise in RIP that may lead to increased renal intratubular pressure. This rise in intratubular pressure may be sufficient to oppose the intraglomerular hydrostatic pressure, resulting in a reduction in GFR. This situation might be analogous to the reduction in GFR observed in obstructive uropathy.^14^

Lastly, human abdominal compression studies by Bradley et al. in 1947 revealed a 24.4% and 27.5% reduction in RBF and GFR and a concomitant increase in RVP to about 20 mm Hg.^15^ Several years later, however, Blake et al. found that RPF, GFR, and filtration fraction (the ratio of GFR to RPF) did not change significantly since RVP was increased from 7.4 mm Hg to 25 mm Hg.^16^ In these particular studies, as RVP was raised, renal arterial pressure was held constant; therefore, the AV gradient decreased but the RPF remained unchanged. Blake concluded that a decrease in resistance occurred somewhere in the renal circuit, most likely distal to the glomerulus (because neither the GFR nor the filtration fraction changed). When RVP was increased further to about 40 mm Hg, a decrease in GFR and RPF were noted, presumably due to the autoregulatory mechanism being overwhelmed. In contrast, Selkurt et al. found that an increase in RVP from 7.5 mm Hg to 22.4 mm Hg in dogs led to a decline in RBF and creatinine clearance but found no change in filtration fraction (FF).^17^ It was postulated that the decrease in RBF and clearance must be due to a decrease in AV gradient, caused by the elevation in RVP since mean arterial pressure was held constant.

### Venous Congestion Mechanisms Leading to Reduction in GFR

**Figure 1:**
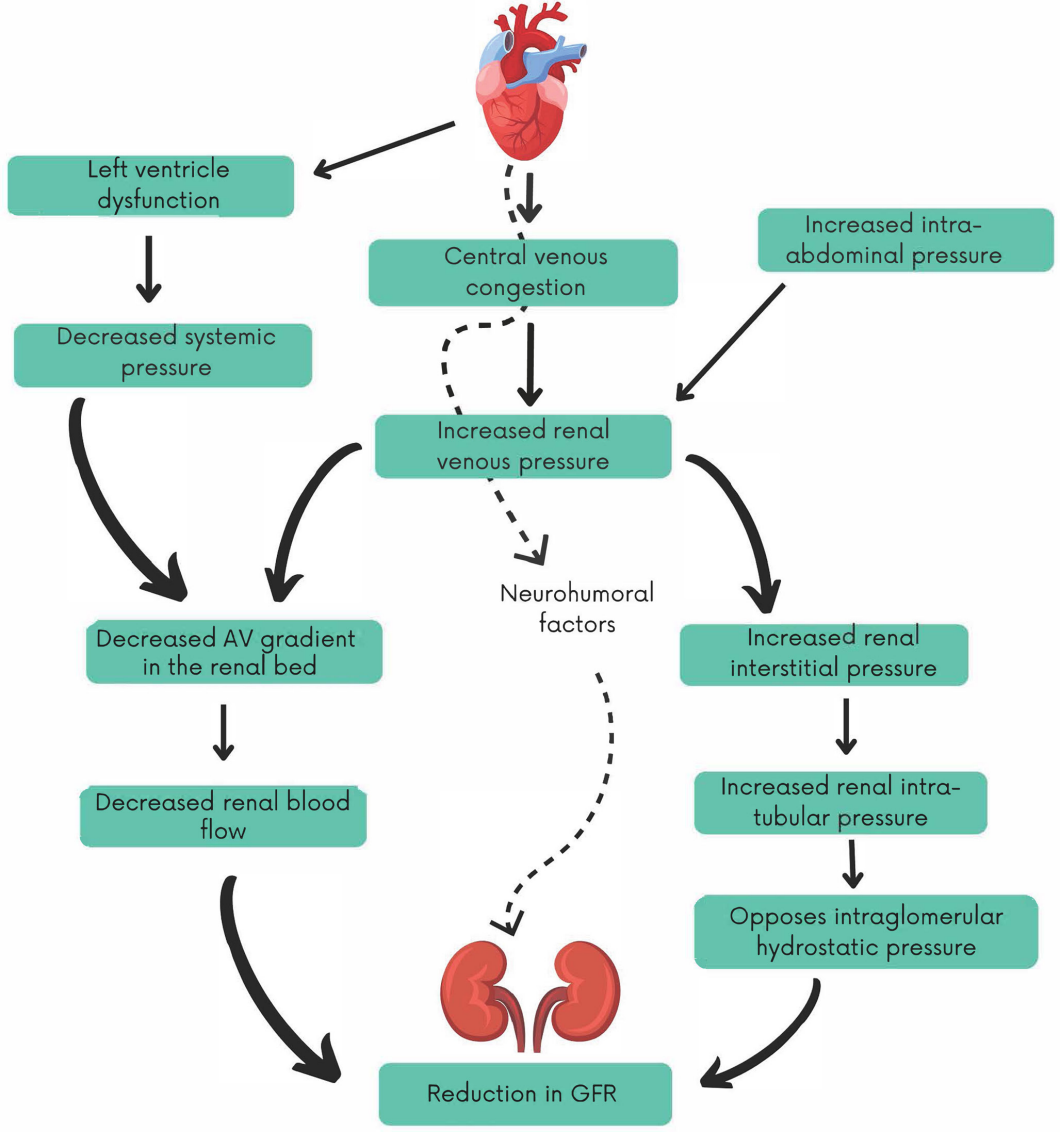
Venous congestion and reduced glomerular filtration rate. AV: arteriovenous; GFR: glomerular filtration rate

To address how increments in RVP can alter GFR, Kishimoto et al. investigated RBF and GFR in response to an elevation in RVP related to renin secretion and distribution of intrarenal blood flow.^18^ They found that neither RBF nor GFR declined significantly until RVP was increased to 30 mm Hg. Nevertheless, at RVP below 30 mm Hg, renin secretion increased and intrarenal blood flow patterns changed with redistribution from the outer to the inner cortex. The following year, the same research group confirmed a similar redistribution of cortical blood flow to the inner cortex when RVP was increased to 44 mm Hg.^19^ Following Kishimoto’s observation that renin secretion increases with elevated RVP, Kastner et al. investigated the role of elevated RVP on the individual components of the renin-angiotensin-aldosterone system.^20^ RBF, GFR, FF, and total renal resistance (TRR) were assessed with and without infusion of an angiotensin-converting enzyme inhibitor (ACEI), while RVP was elevated to 50 mm Hg in dogs. The control group showed no significant change in RBF, GFR, or FF, but TRR declined in a linear fashion as RVP rose. Renin secretion increased modestly as RVP rose to 30 mm Hg but increased markedly at higher pressures, as demonstrated by Abe et al. Generally, at RVP above 20 mm Hg to 30 mm Hg, dogs infused with an ACEI showed a decline in GFR, FF, and TRR. As RVP was increased, GFR and FF in those infused with ACEI remained significantly lower than control dogs.^19^ Figure 1 depicts the central alterations that may conspire to lead to a lower GFR in the setting of heart failure.

## Issues in Volume Assessment

With the emergence of point-of-care ultrasound (POCUS), the clinician now has an additional tool to help estimate the degree of venous congestion without the need for a central venous catheter. Beaubien-Souligny et al. developed a protocolized venous Doppler examination termed “venous excess ultrasound score” (VExUS).^21^ A cohort of 145 patients undergoing cardiac surgery with cardiopulmonary bypass underwent doppler interrogation with POCUS, including the hepatic vein, portal vein, intrarenal vein, and measurement of inferior vena cava diameter. Five different prototypes of grading systems were compared; all were assigned a numeric value from 0 to 3 based on different sonographic criteria. VExUS was obtained before surgery and up to the third postoperative day. They were able to predict the development of acute kidney injury in 96% of the patients with a VExUS prototype C Grade 3 score, and this approach outperformed single CVP measurements with a central venous catheter.^21^ Multipoint Doppler interrogation with POCUS for volume assessment has gained the attention of the nephrology community, not only to help detect signs of venous congestion but also to assess restoration of the RBF in hypoperfused states.^22^

### Volume Control

Hypervolemia is a core target for therapy in heart failure. Diuretics are essential for volume control: although their effect on mortality and morbidity has not been formally studied with randomized controlled trials, their clinical usefulness is indisputable. Thiazide and thiazide-like diuretics are less potent than loop diuretics because they need to be filtered and secreted to exert their diuretic effect and thereby may lose some effect as renal function declines. It is thought that inhibition of the Na-K-2Cl channel on the apical membrane of the ascending loop of Henle with loop diuretics preserves the linear relationship between natriuresis and GFR. Therefore, thiazide diuretics have not traditionally been recommended when GFR < 30 mL/min/1.73^2^.^23^ Contemporary evidence, however, shows that this may not be the case. In a double-blind prospective controlled trial of 2,849 patients with GFR < 30 mL/min/1.73^2^ who were randomized to either receive chlorthalidone or placebo for blood pressure control, the chlorthalidone group not only achieved a 15.7 mm Hg reduction in blood pressure but also had reductions in body weight, body volume, N-terminal pro–B-type natriuretic peptide, and an increase in plasma renin and aldosterone levels—all consistent with a robust pharmacological effect of diuresis even at this GFR level.^24^

### Diuretic Resistance

Furosemide, bumetanide, and torsemide are the most commonly used loop diuretics, with 40 mg of furosemide equivalent to 20 mg of torsemide and 1 mg of bumetanide. Furosemide has the most erratic oral bioavailability of the three. A subset of patients with heart failure fail to respond to loop diuretics, and the term “diuretic resistance” (DR) is used in the literature to describe this phenomenon. The mechanism classically considered to cause DR is distal tubule hypertrophy and hyperfunction causing rebound sodium reabsorption.^25,26^ It is important to distinguish this from the braking phenomenon described in healthy volunteers using loop diuretics, which is far from pathological, necessary to preserve homeostasis, and prevents lethal sodium excretions.^27^

Patients who do not respond to furosemide are sometimes put on a different loop diuretic. Another approach is changing the administration route from oral to intravenous, especially during acute decompensated heart failure when gastrointestinal absorption of diuretics is altered.^28^ There is no substantial difference in diuresis between continuous infusion and bolus administration of loop diuretics.^29^ For loop diuretics to reach their pharmacological target, they need to be excreted by the organic anion transporter-1 and are highly bound to serum albumin (> 95%), so they are minimally filtered.^30^ Administering mixtures of albumin and loop diuretics was suggested as a strategy to overcome diuretic resistance in hypoalbuminemic patients by theoretically increasing the amount of diuretic available for tubular secretion. This practice is not supported by evidence, as multiple studies have shown that addition of albumin to a furosemide infusion does not enhance diuresis but certainly adds to the already costly admission of a heart failure episode.^31,32,33^

Combination of loop diuretics and thiazides is an effective approach to overcome DR. This also has been referred to as a sequential nephron blockade. By blocking the distal tubule sodium reabsorption, thiazides use can alleviate the hypertrophy and hyperfunctioning caused by loop diuretic therapy and potentially may overcome DR.^26^

### Ultrafiltration

Using the standard hemodialysis apparatus and bypassing the dialysate step, one can remove isotonic fluid from the circulation. This is referred to as “ultrafiltration”—quite a popular approach years ago but the practice decreased after results of the UNLOAD trial showed that it was not more helpful than diuretics. This was a large prospective nonblinded randomized trial that enrolled a total of 200 patients to intravenous diuretics or ultrafiltration.^34^ While patients in the ultrafiltration arm had greater weight loss and reduced 90-day rehospitalization rate, they encountered a higher rate of adverse events and a similar change in serum creatinine and dyspnea scores. The diuretic arm received an average daily furosemide-equivalent dose of 181 mg. One can make the argument that adding a thiazide or increasing the loop diuretic dose would have achieved similar 90-day rehospitalization rates.

## Conclusion

The heart and kidney commonly share risk factors for organ dysfunction, and a sequential bidirectional causal relationship between the two organs has already been established. Elevated central venous pressure is undoubtedly responsible for backward failure. Loop diuretics are the first-line treatment for decongestive therapy, and diuretic resistance can be overcome by changing to another loop diuretic, changing from oral to intravenous administration, or adding a thiazide diuretic. Adding albumin admixtures to loop diuretic therapy does not enhance diuresis, and continuous intravenous administration has essentially the same diuretic effect as a bolus administration. Collectively, a team approach is essential in the care of these patients.

## Key Points

- Elevated central venous pressure leads to a lower arteriovenous gradient across the renal bed, which results in reduced renal blood flow and glomerular filtration rate (GFR).
- Elevated central venous pressure is transmitted back to the renal interstitial space and subsequently to the renal tubules, thus opposing intraglomerular hydrostatic pressure with a resultant decrease in GFR.
- Diuretic resistance is caused by distal tubule hypertrophy and hyperfunction, causing both rebound sodium reabsorption.
- Sequential nephron blockade ameliorates distal nephron hypertrophy and can be used to overcome diuretic resistance.
- Intravenous and bolus administration of loop diuretics are equivalent, and there is no benefit in adding albumin admixtures to loop diuretics in hypoalbuminemia patients.
